# Supplementary material for: Use and Evaluation of a pES213-Derived Plasmid for the Constitutive Expression of gfp Protein in Pathogenic Vibrios: a Tagging Tool for In Vitro Studies
Source: Microbiol Spectr. 2022 Dec 12;11(1):e02490-22. doi: 10.1128/spectrum.02490-22 (PMC9927583; doi:10.1128/spectrum.02490-22)
Supplement: Supplemental file 1 — Supplemental material. Download spectrum.02490-22-s0001.pdf, PDF file, 1.1 MB [file spectrum.02490-22-s0001.pdf]

Table S1: pVSV102 conjugation efficacy for all target vibrios. Mixtures mated triparentally with E. coli carrying the helper plasmid pEVS104 and the donor plasmid pVSV102 on kanamycin amended media equivalent to the designated stress concentration. Mating mixtures were performed on LBS 3% agar plates amended with 300 µg mL^{-1} kanamycin and incubated at 28 ^{o}C for up to 72 h.

Species	Strain	Kanamycin Stress Concentration (µg mL^{-1})	Number of Mating Mixtures Attempted	CFU of Target Vibrio Added[a]	Development of Fluorescent Patches[b] (hours)	Number of Successful Mating Mixtures
Photobacterium damselae	ATCC 33539	15	24	$3.8x10^{7}$	NA[c]	24
Vibrio alginolyticus	ATCC 17749	75	24	$4.6x10^{7}$	24	21
Vibrio anguillarum	ATCC 19264	35	24	$3.0x10^{7}$	48	24
Vibrio campbellii	ATCC 25920	35	24	$6.3x10^{7}$	24	24
Vibrio coralliilyticus	ATCC BAA-450	50	24	$3.3x10^{4}$	72	24
Vibrio cholerae	ATCC 14035	50	24	$2.9x10^{6}$	48	20
Vibrio harveyi	ATCC 14126	25	24	$7.1x10^{6}$	72[c]	16
Vibrio mediterranei	ATCC 43341	100	24	$2.3x10^{7}$	24	24
Vibrio parahaemolyticus	ATCC 43996	35	24	$3.7x10^{7}$	24	24
Vibrio pelagius	ATCC 25916	25	24	$4.4x10^{5}$	48	11
Vibrio splendidus	ATCC 33869	75	24	$1.16x10^{7}$	48	10
Vibrio vulnificus	ATCC 27562	50	24	$6.2x10^{7}$	48	24

[a]All mating mixtures contained 6.0x10^6 CFU of *E. coli* carrying the helper plasmid pEVS104, 6.3x10^6 CFU of *E. coli* carrying the donor plasmid pVSV102, and the target vibrio indicated in this column.
[b]The development of fluorescent patches suggests that the mating mixture resulted in a successful conjugation trial. Observation of these patches can be used as a laboratory diagnostic tool to indicate potentially successful mixtures to progress to purification. Confirmation of the purified vibrio must be accomplished using fluorescent microscope.
[c]Fluorescence is not visible in the gross morphology of *P. damselae* mating mixtures and is not always visible in *V. harveyi* mating mixtures. Conjugation success for these species must be identified via microscopy following purification of the mixture on TCBS agar.

Table S2: Evaluation of interspecies mobilization of pVSV102 from tagged *V. parahaemolyticus* into non-tagged *V. cholerae* and *V. vulnificus* grown in co-culture in antibiotic-free media. Species identity confirmed using CHROMagar *Vibrio*. Fluorescence identified using gross observation of the CFU illuminated by a 495 nm blacklight and confirmed with fluorescent microscopy (495 excitation wavelength).

Time (hours)	Total V. parahaemolyticus (CFU)	Fluorescent V. parahaemolyticus (CFU)	Non-Fluorescent V. parahaemolyticus (CFU)	Total V. cholerae or V. vulnificus (CFU)	Total Fluorescent V. cholerae or V. vulnificus (CFU)	Total Non-Fluorescent V. cholerae or V. vulnificus (CFU)
0	28	28	0	21	0	21
24	8	8	0	45	0	45
48	8	8	0	35	0	35
72	4	4	0	40	0	40
96	5	4	1	73	0	73
120	7	5	2	90	0	90

Supplemental Material
Norfolk & Lipp, 2022

Table S3: Maximum observed mean GFP loss for all tagged vibrio strains. Antibiotic supplemented cultures maintained at 300µg/mL kanamycin.

Species	Strain	Time (hours)[a]	Media Type	Antibiotics Present (+ or -)	Percent of CFU that Lost Fluorescence (%)[b]
Photobacterium damselae	ATCC 33539	120	LBS 3%	-	3.4
				+	0.0
		48	ASW	-	3.0
Vibrio alginolyticus	ATCC 17749	120	LBS 3%	-	2.0
				+	0.0
		48	ASW	-	0.0
Vibrio anguillarum	ATCC 19264	48	LBS 3%	-	18.5
		120		+	0.0
		48	ASW	-	0.0
Vibrio campbellii	ATCC 25920	96	LBS 3%	-	5.6
		120		+	0.0
		48	ASW	-	0.0
Vibrio coralliilyticus	ATCC BAA-450	120	LBS 3%	-	33.3
				+	0.0
		48	ASW	-	3.8
Vibrio cholerae	ATCC 14035	120	LBS 3%	-	90.0
				+	0.0
		48	ASW	-	9.8
Vibrio harveyi	ATCC 14126	120	LBS 3%	-	37.1
				+	0.0
		24	ASW	-	8.7
Vibrio mediterranei	ATCC 43341	72	LBS 3%	-	27.3
		96		+	10.3
		48	ASW	-	1.7
Vibrio parahaemolyticus	ATCC 43996	72	LBS 3%	-	7.9
		120		+	0.0
		48	ASW	-	0.0
Vibrio pelagius	ATCC 25916	120	LBS 3%	-	11.1
				+	0.0[*]
		0	ASW	-	0.0
Vibrio splendidus	ATCC 33869	120	LBS 3%	-	11.4
				+	0.0
		24	ASW	-	0.0[*]
Vibrio vulnificus	ATCC 27562	72	LBS 3%	-	10.9
		120		+	0.0
		48	ASW	-	18.4

[a]Indicates the timepoint at which each transconjugant vibrio demonstrated the greatest loss of GFP.
[b]A loss value of 0.0% indicates that no CFU lost fluorescence under the described culture conditions. Values denotes with a * symbol indicate cultures that were not recoverable throughout the entirety of the experiment.

Table S4: Generation estimation, CFU concentrations, and optical density for subculture experimentation. Generations calculated as the number of number of doubling cycles required for the inoculation CFU to surpass the stationary phase CFU rounded to the nearest whole generation.

Species	Strain	CFU Inoculated Per Subculture (100 µL)	Total CFU at Stationary Phase (4 mL)	OD_{600} After Inoculation (0 h)	OD_{600} at Stationary Phase (~18 h)	Estimated Generations to Stationary Phase
Photobacterium damselae	ATCC 33539	$4.2x10^{7}$	$6.8x10^{8}$	0.046	1.647	6
Vibrio alginolyticus	ATCC 17749	$1.4x10^{7}$	$8.8x10^{8}$	0.099	2.006	5
Vibrio anguillarum	ATCC 19264	$3.5x10^{7}$	$1.2x10^{9}$	0.054	1.835	6
Vibrio campbellii	ATCC 25920	$6.7x10^{7}$	$1.84x10^{9}$	0.065	1.434	5
Vibrio coralliilyticus	ATCC BAA-450	$4.0x10^{7}$	$1.64x10^{8}$	0.070	1.371	5
Vibrio cholerae	ATCC 14035	$1.6x10^{7}$	$2.08x10^{8}$	0.075	1.741	5
Vibrio harveyi	ATCC 14126	$2.6x10^{7}$	$1.12x10^{8}$	0.041	1.514	6
Vibrio mediterranei	ATCC 43341	$2.4x10^{7}$	$1.56x10^{9}$	0.050	1.557	5
Vibrio parahaemolyticus	ATCC 43996	$3.7x10^{7}$	$2.32x10^{9}$	0.067	1.824	5
Vibrio pelagius	ATCC 25916	$1.7x10^{6}$	$1.32x10^{7}$	0.031	1.361	6
Vibrio splendidus	ATCC 33869	$1.18x10^{7}$	$1.08x10^{8}$	0.044	1.564	6
Vibrio vulnificus	ATCC 27562	$4.6x10^{7}$	$2.44x10^{9}$	0.052	1.636	5

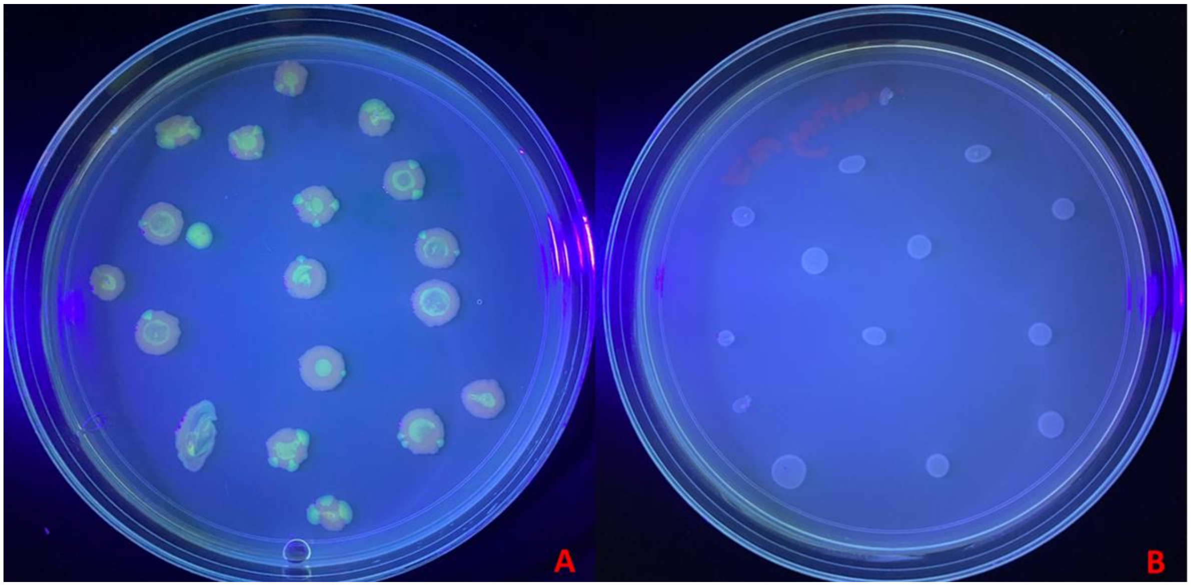

Figure S1: GFP mating mixtures of *V. mediterranei* illuminated with the aid of a 495 nm blacklight. (A) Mating mixture demonstrating potentially successful conjugation of the *gfp*. Bright green patches within the cell masses indicate successful transfer trials that can be moved to the purification (TCBS streaking) step. (B) Unsuccessful mating mixture with no visible fluorescence. The unsuccessful result of this mixture was due to the use of a kanamycin concentration (150 µg mL^{-1}) that was above the tolerance limit for *V. mediterranei* which likely resulted in death of the cells before conjugation could be accomplished. It should be noted that while this visible check was useful in the transfer of pVSV102, this method my not be amenable to other more subtle reporters.

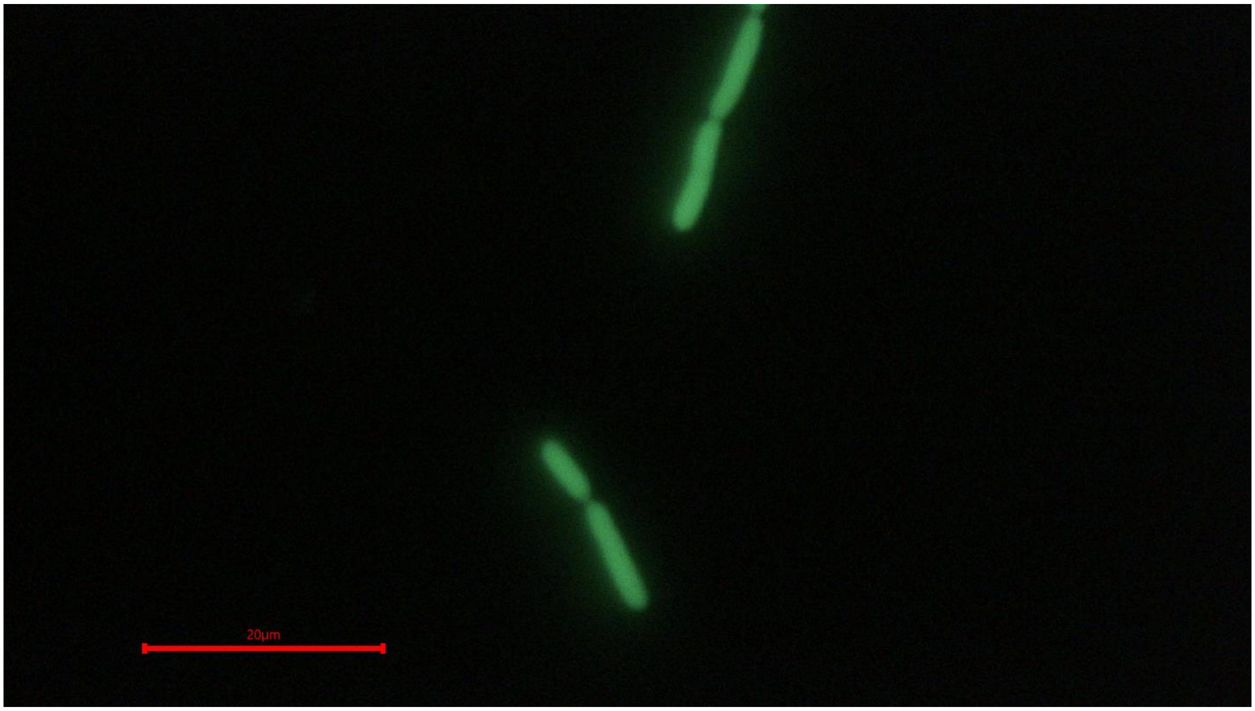

Figure S2: GFP tagged *P. damselae*. Culture grown from a -80°C frozen stock of the purified GFP-tagged culture. Image taken after overnight growth in LBS 3% amended with 300µg/mL kanamycin. Source strain ATCC 33539. Magnification at 1000X.

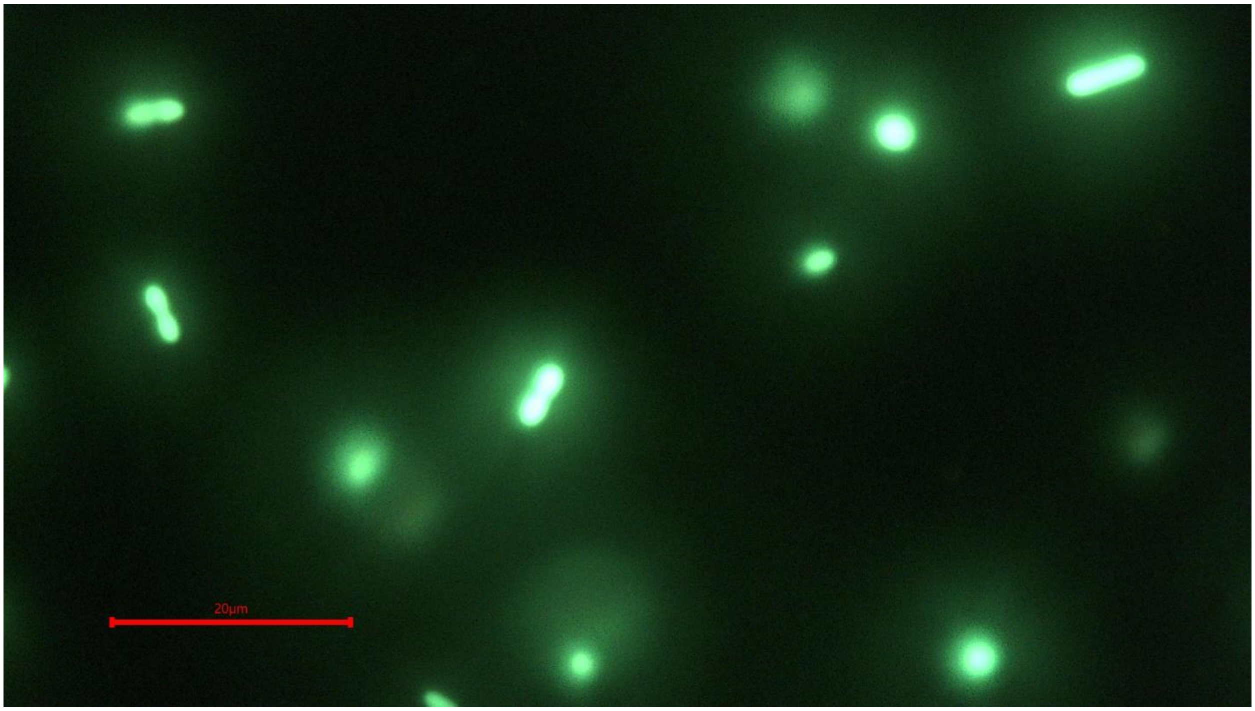

Figure S3: GFP tagged *V. alginolyticus*. Culture grown from a -80°C frozen stock of the purified GFP-tagged culture. Image taken after overnight growth in LBS 3% amended with 300µg/mL kanamycin. Source strain ATCC 17749. Magnification at 1000X.

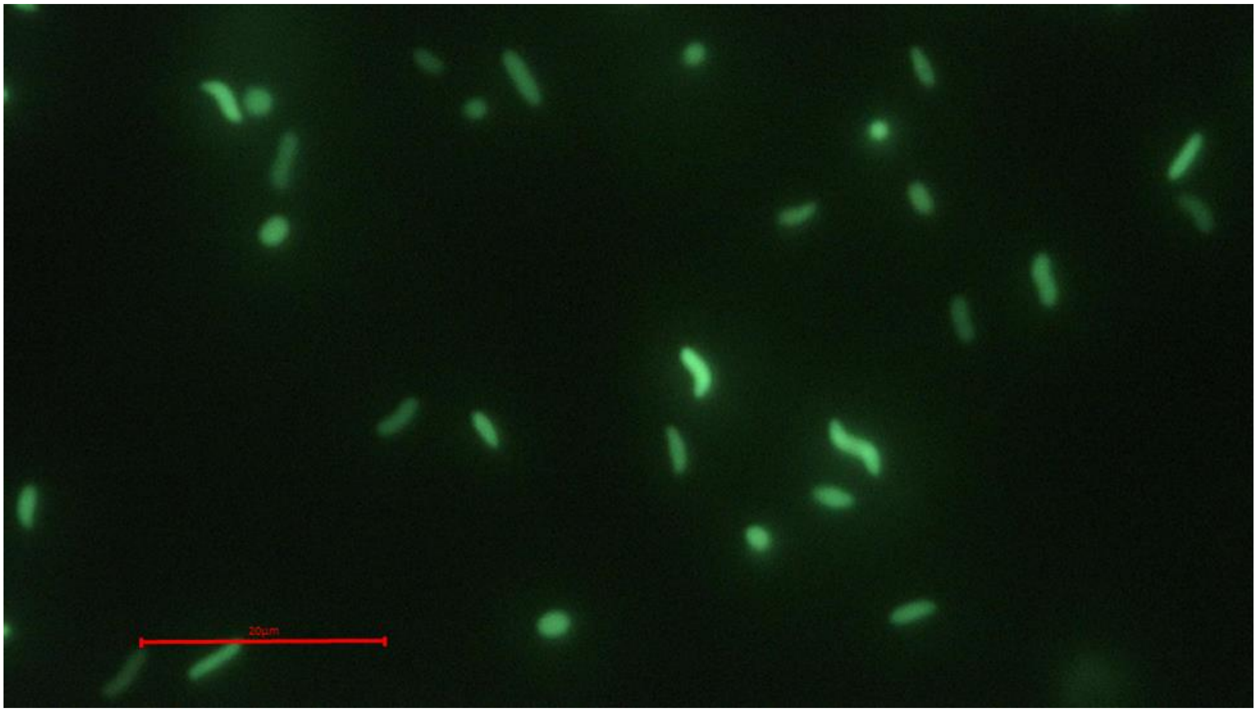

Figure S4: GFP tagged *V. anguillarum*. Culture grown from a -80°C frozen stock of the purified GFP-tagged culture. Image taken after overnight growth in LBS 3% amended with 300µg/mL kanamycin. Source strain ATCC 19264. Magnification at 1000X.

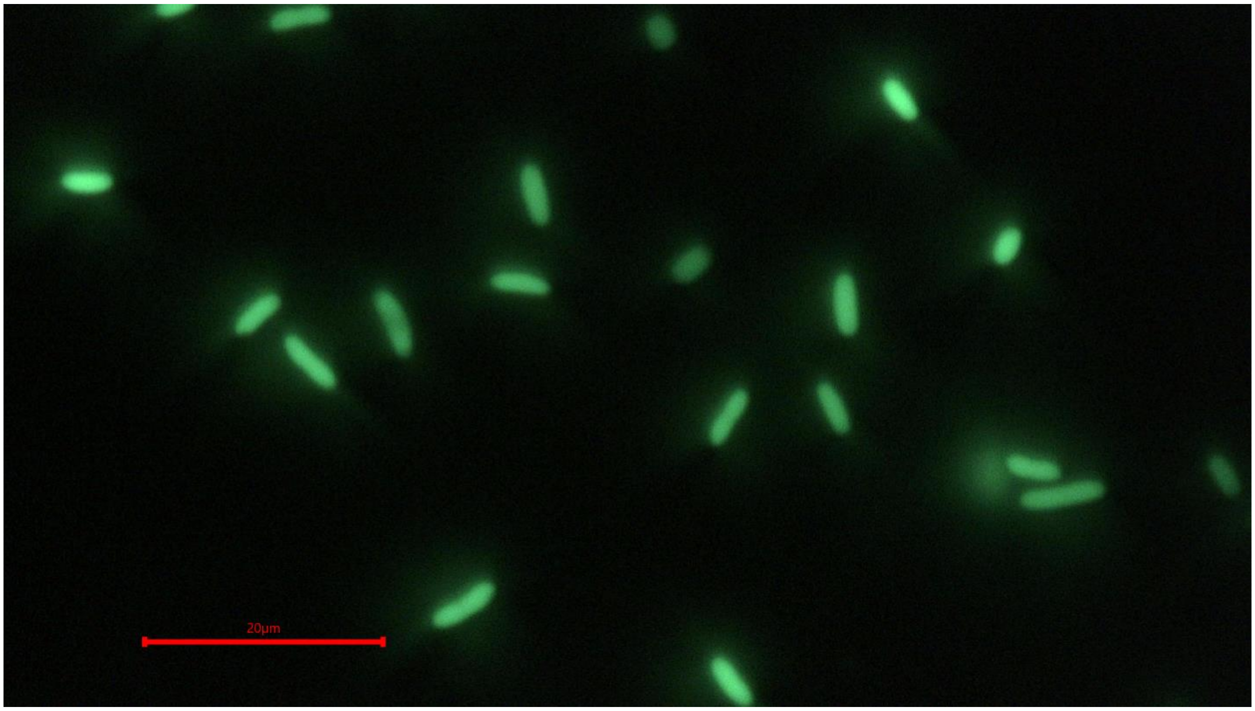

Figure S5: GFP tagged *V. campbellii*. Culture grown from a -80°C frozen stock of the purified GFP-tagged culture. Image taken after overnight growth in LBS 3% amended with 300µg/mL kanamycin. Source strain ATCC 25920. Magnification at 1000X.

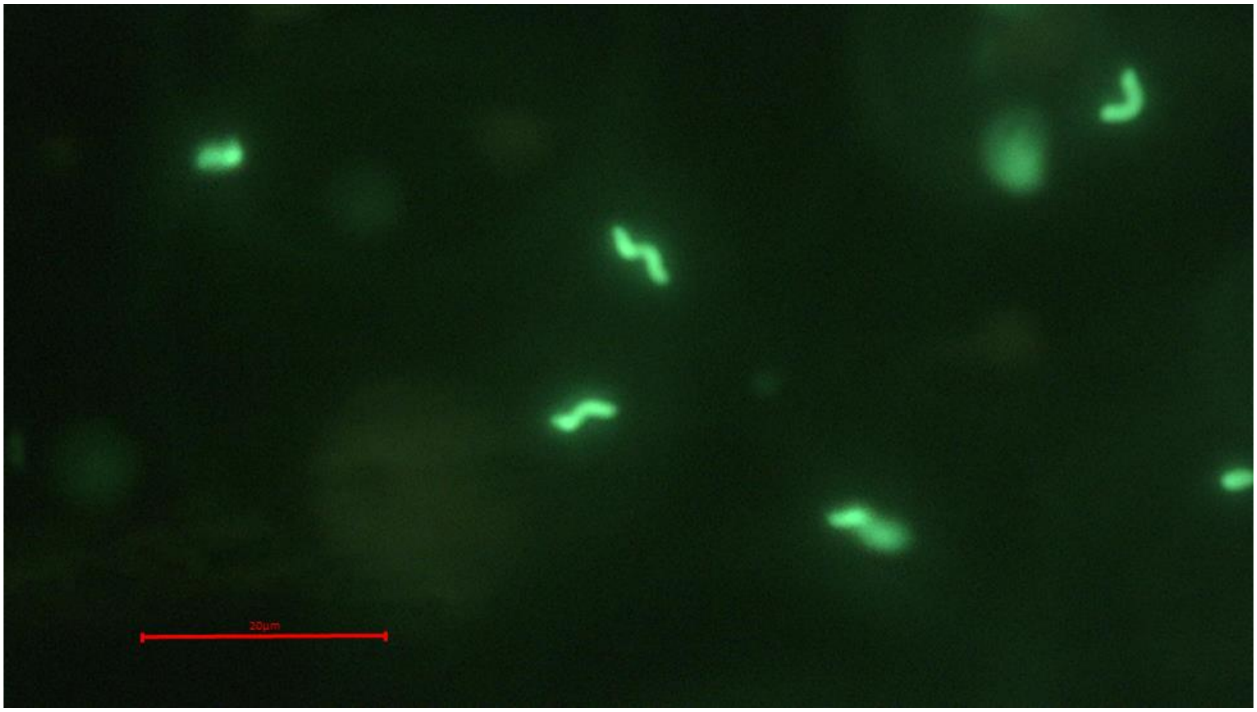

Figure S6: GFP tagged *V. coralliilyticus*. Culture grown from a -80°C frozen stock of the purified GFP-tagged culture. Image taken after overnight growth in LBS 3% amended with 300µg/mL kanamycin. Source strain ATCC BAA-450 Magnification at 1000X.

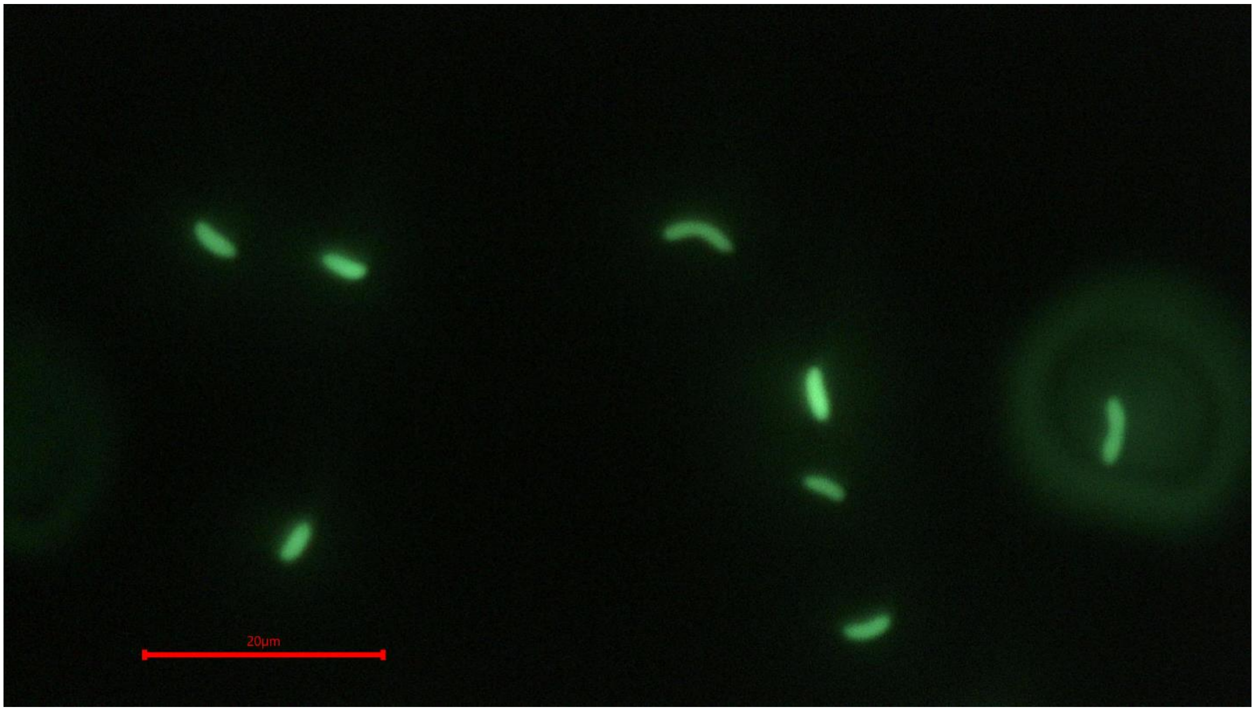

Figure S7: GFP tagged *V. cholerae*. Culture grown from a -80°C frozen stock of the purified GFP-tagged culture. Image taken after overnight growth in LBS 3% amended with 300µg/mL kanamycin. Source strain ATCC 14035. Magnification at 1000X.

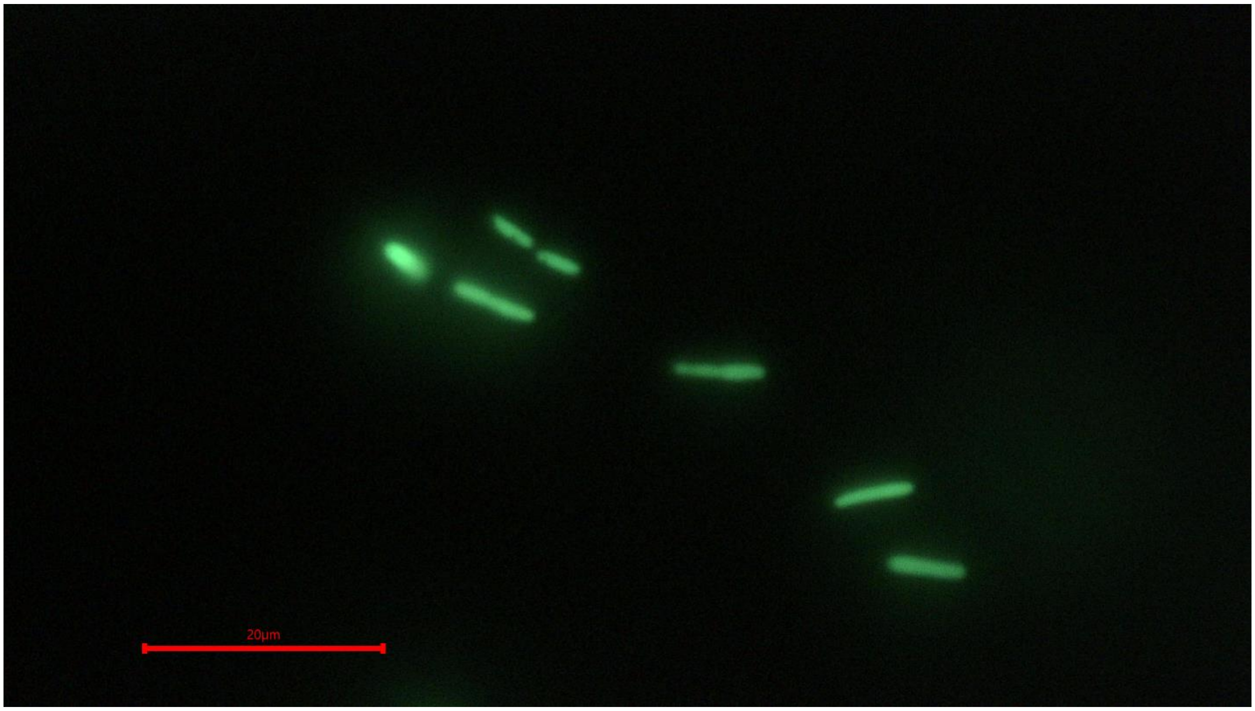

Figure S8: GFP tagged *V. harveyi*. Culture grown from a -80°C frozen stock of the purified GFP-tagged culture. Image taken after overnight growth in LBS 3% amended with 300µg/mL kanamycin. Source strain ATCC 14126. Magnification at 1000X.

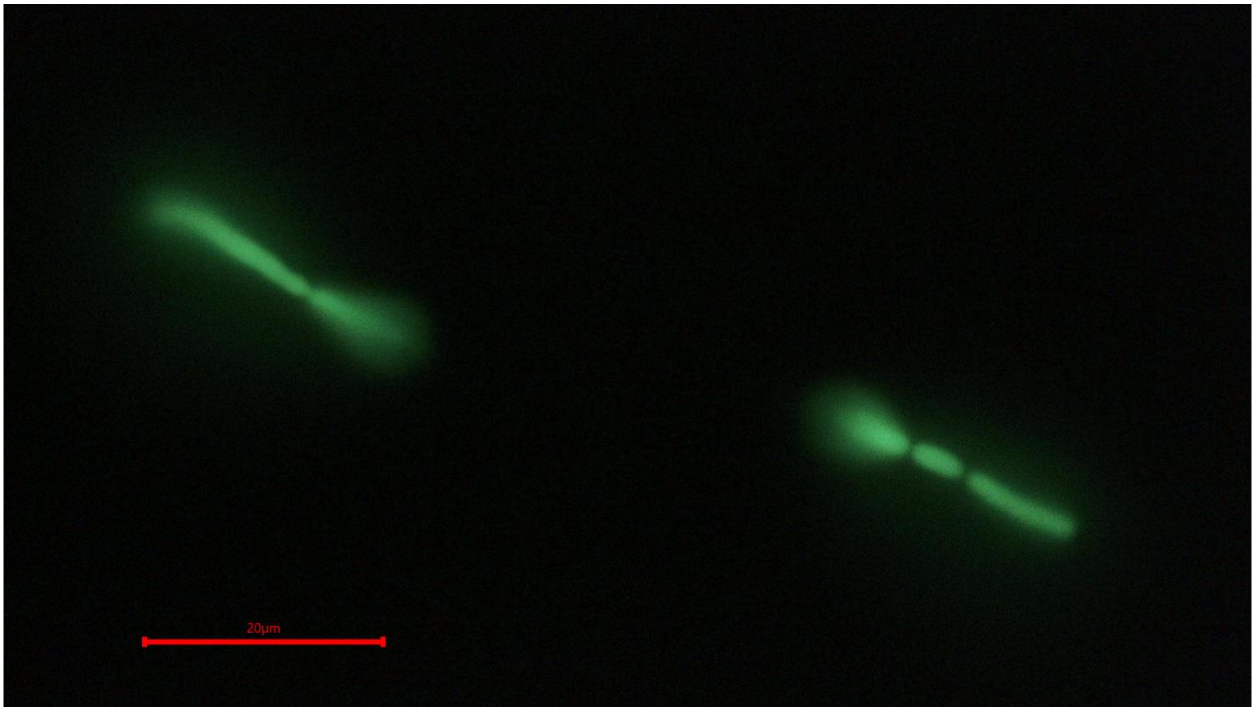

Figure S9: GFP tagged *V. mediterranei*. Culture grown from a -80°C frozen stock of the purified GFP-tagged culture. Image taken after overnight growth in LBS 3% amended with 300µg/mL kanamycin. Source strain ATCC 43341. Magnification at 1000X.

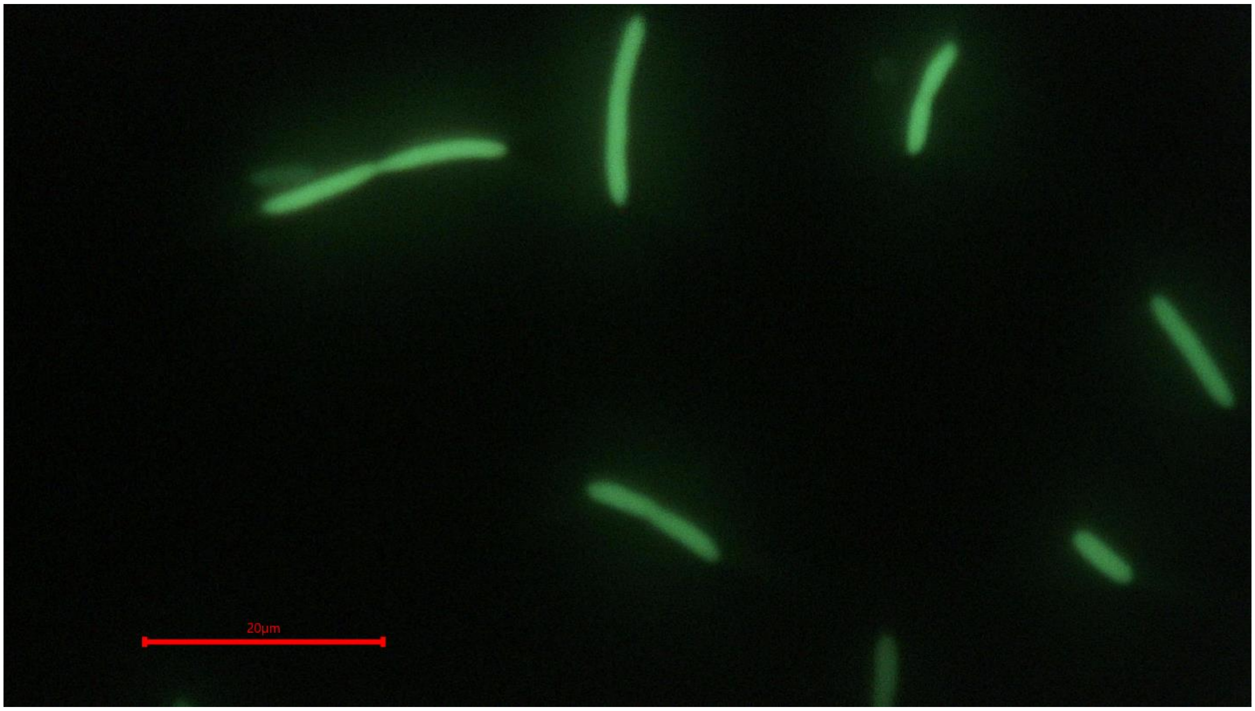

Figure S10: GFP tagged *V. parahaemolyticus*. Culture grown from a -80°C frozen stock of the purified GFP-tagged culture. Image taken after overnight growth in LBS 3% amended with 300µg/mL kanamycin. Source strain ATCC 43996. Magnification at 1000X.

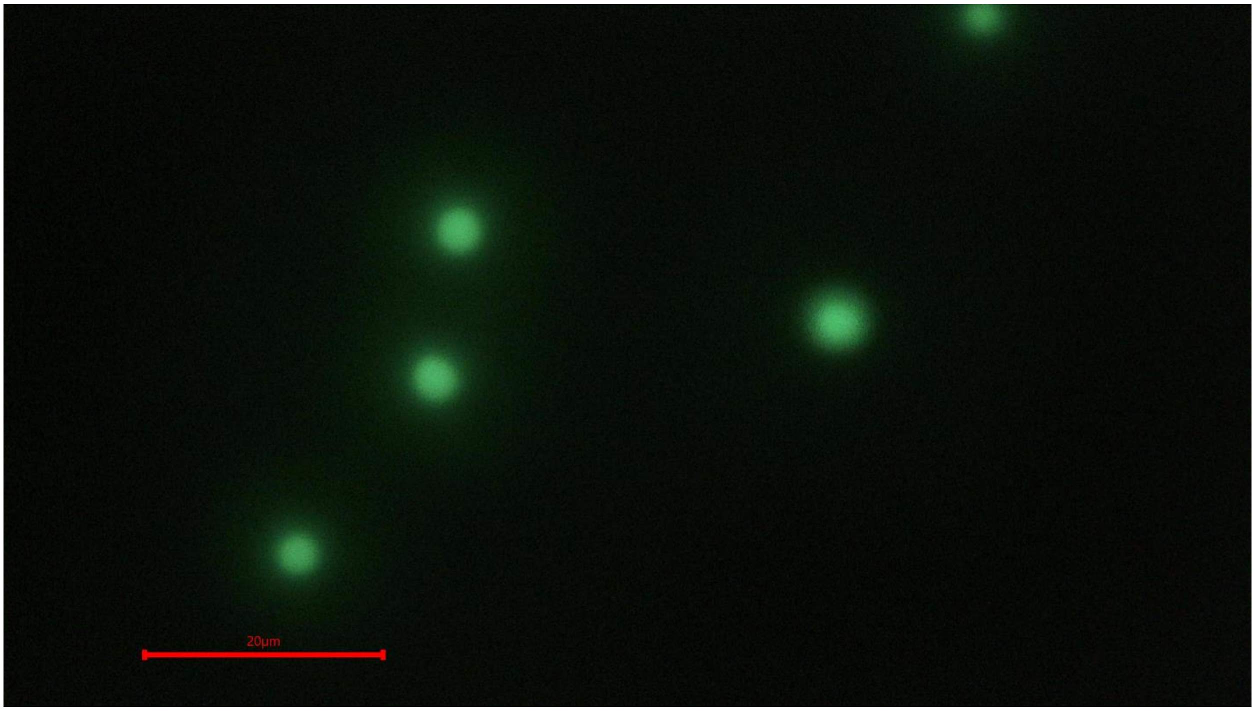

Figure S11: GFP tagged *V. pelagius*. Culture grown from a -80°C frozen stock of the purified GFP-tagged culture. Image taken after overnight growth in LBS 3% amended with 300µg/mL kanamycin. Source strain ATCC 25916. Magnification at 1000X.

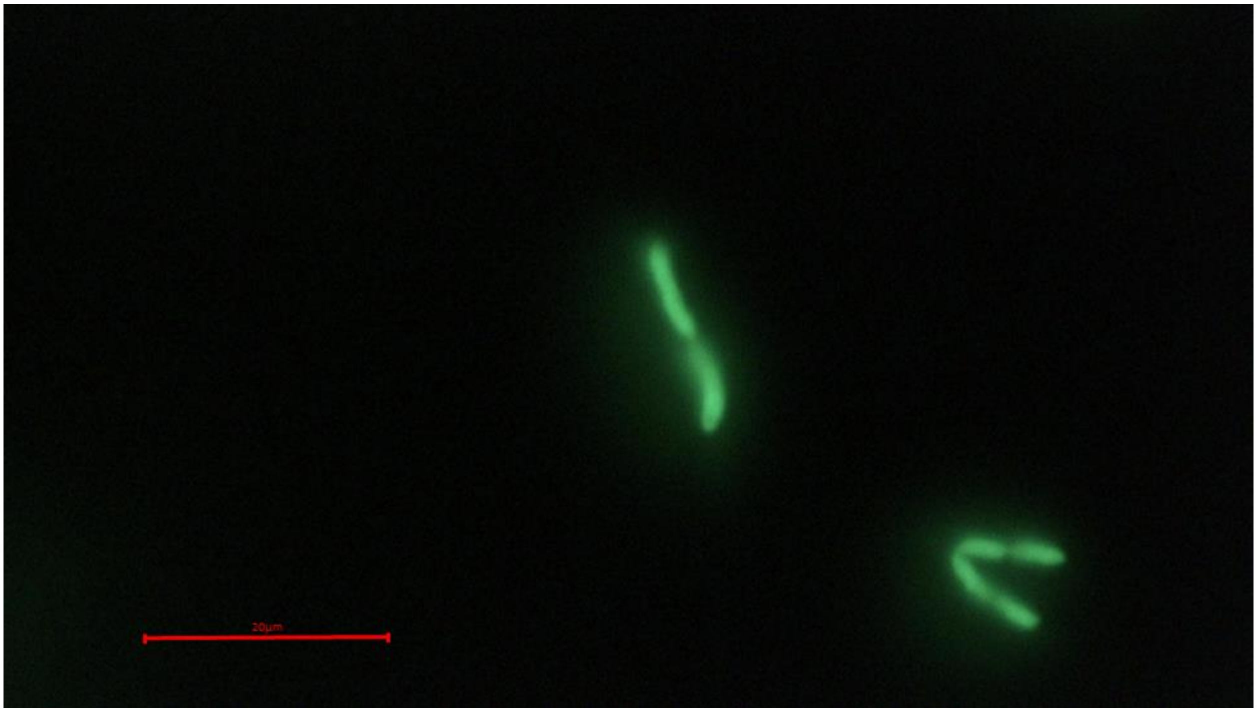

Figure S12: GFP tagged *V. splendidus*. Culture grown from a -80°C frozen stock of the purified GFP-tagged culture. Image taken after overnight growth in LBS 3% amended with 300µg/mL kanamycin. Source strain ATCC 33869. Magnification at 1000X.

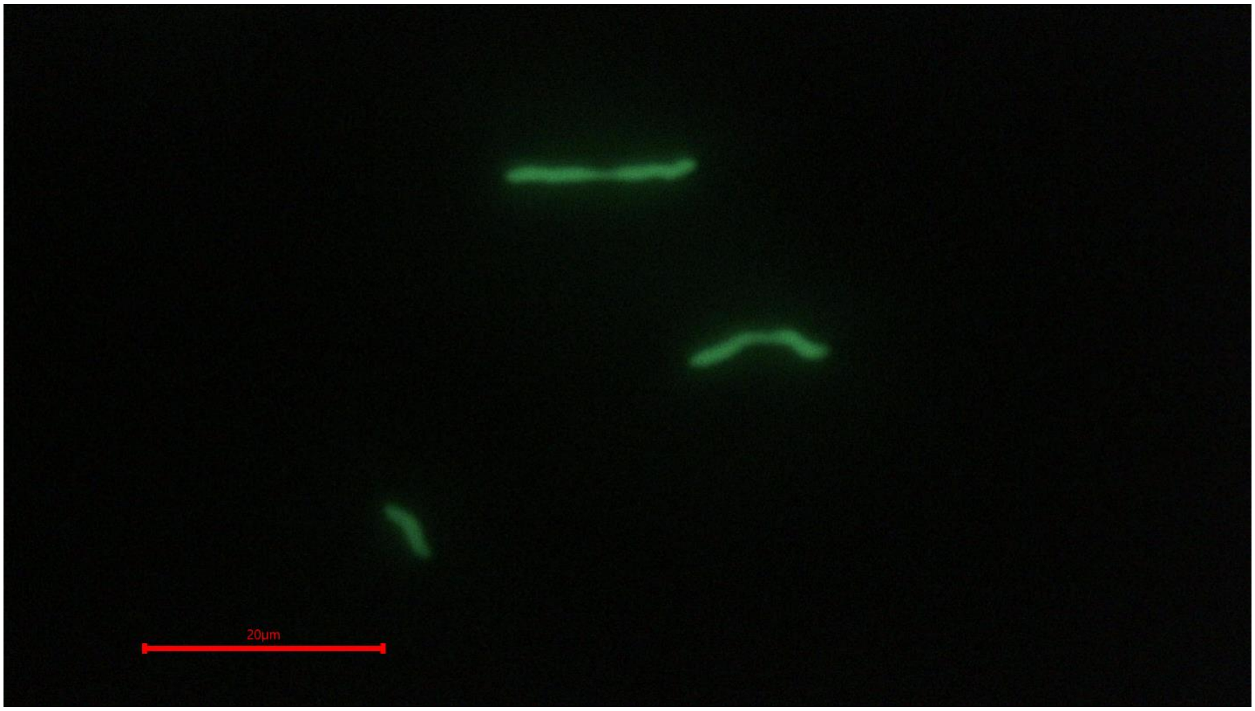

Figure S13: GFP tagged *V. vulnificus*. Culture grown from a -80°C frozen stock of the purified GFP-tagged culture. Image taken after overnight growth in LBS 3% amended with 300µg/mL kanamycin. Source strain ATCC 27562. Magnification at 1000X,

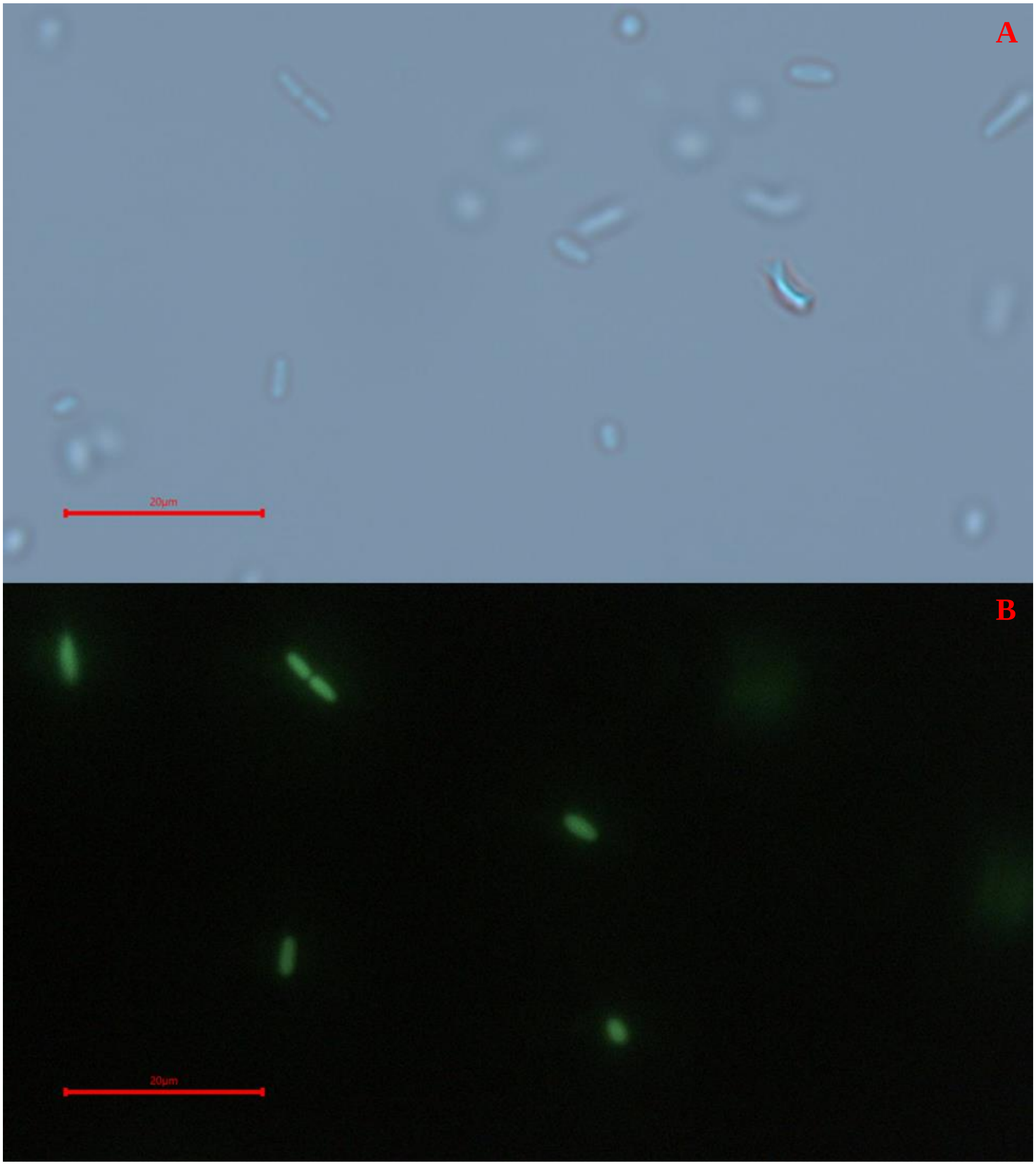

Figure S14: Differentiation of GFP-tagged *V. harveyi* within a complex mixture of vibrios. Mixture contains equal parts *V. alginolyticus* (GFP), *V. campbellii*, *V. parahaemolyticus*, *V. harveyi*, and *V. vulnificus*. Images A and B compare the same micrograph under light microscopy and fluorescent microscopy (495 nm excitation wavelength) at 1000X magnification.

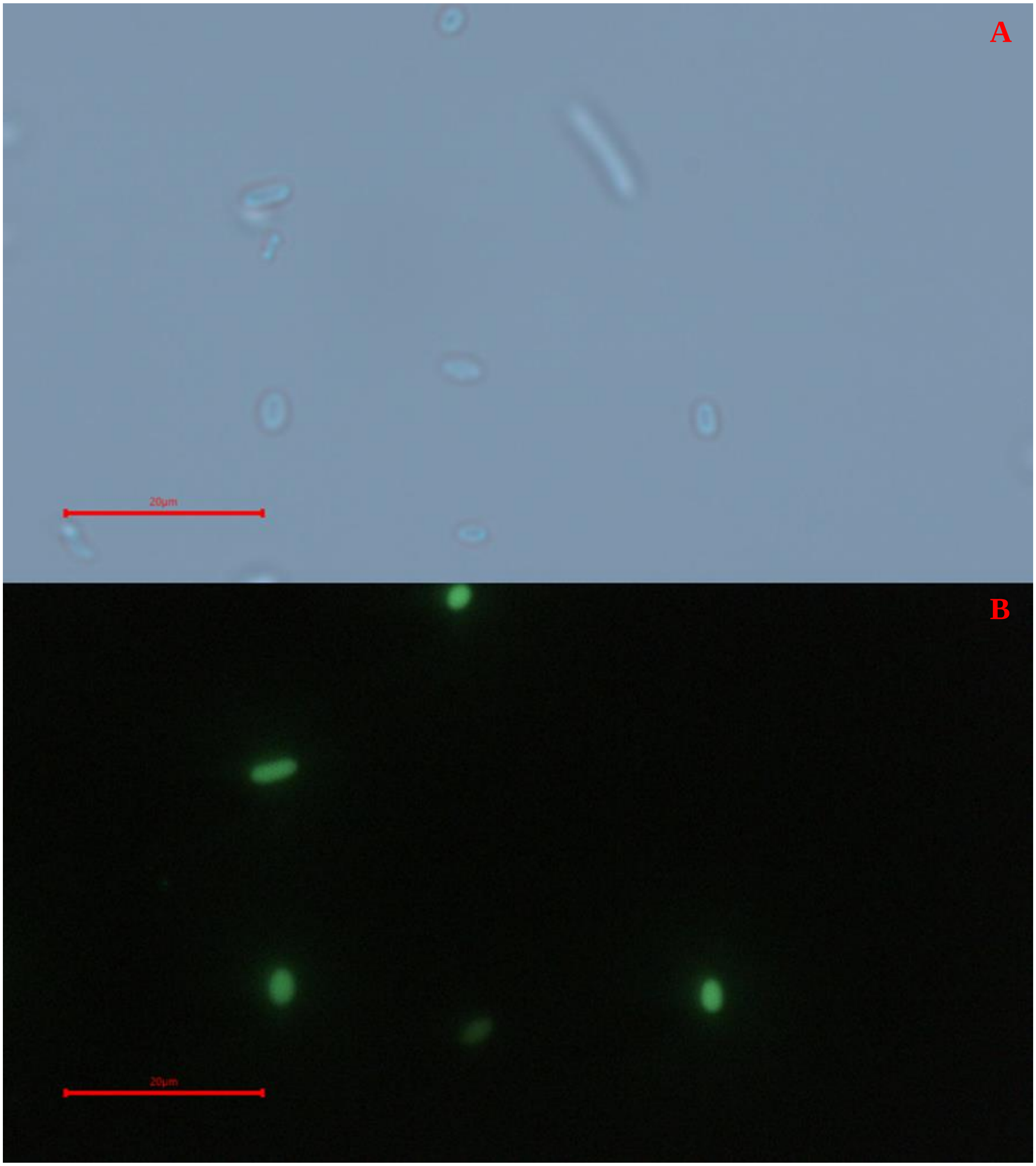

Figure S15: Differentiation of GFP-tagged *V. parahaemolyticus* within a complex mixture of vibrios. Mixture contains equal parts *V. alginolyticus* (GFP), *V. campbellii*, *V. parahaemolyticus*, *V. harveyi*, and *V. vulnificus*. Images A and B compare the same micrograph under light microscopy and fluorescent microscopy (495 nm excitation wavelength) at 1000X magnification.

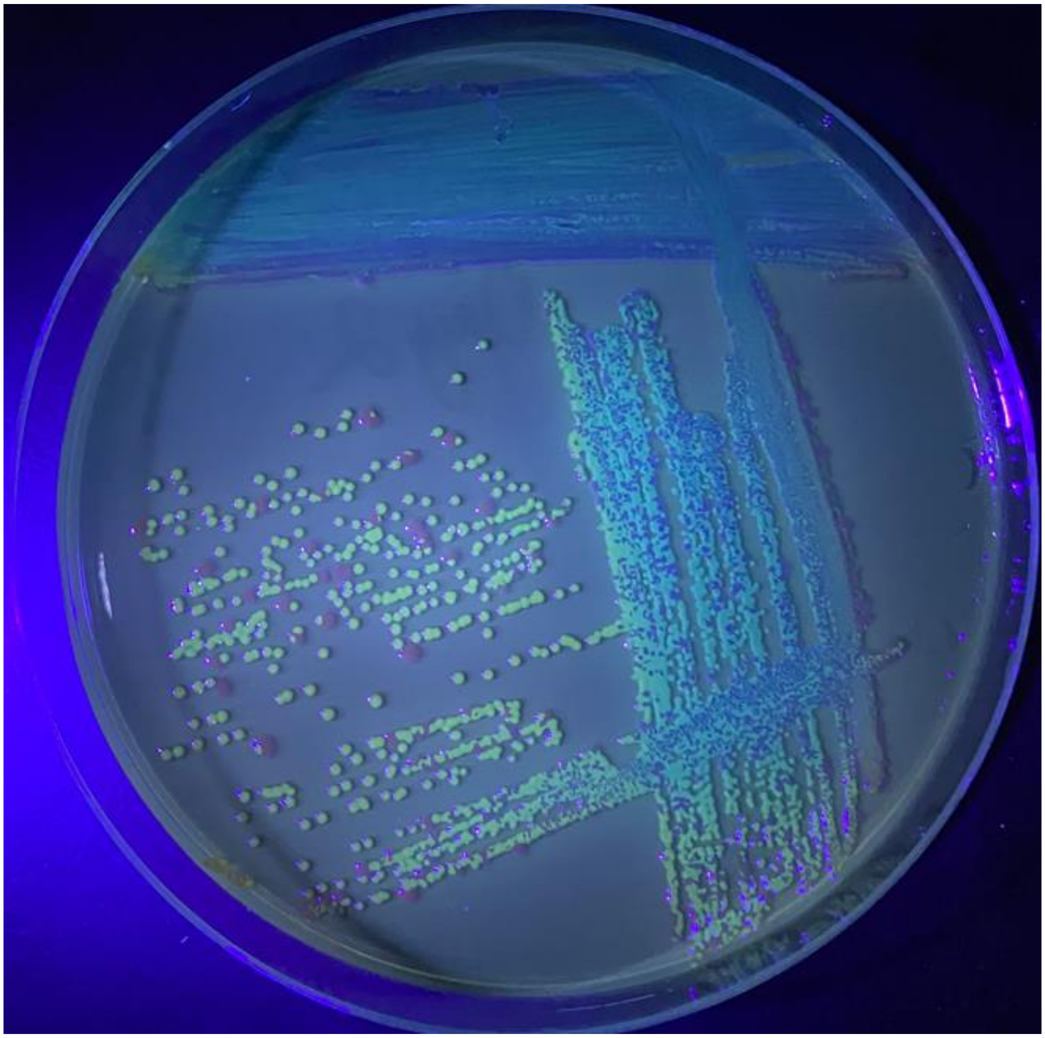

Figure S16: Subculture of *V. cholerae* retention study at T_{48}. Increased colony size was observed in non-fluorescent (purple) colonies compared to fluorescent colonies (green). Image of a100x15mm culture dish with 495 nm blacklight illumination.
